# Adsorption of Antibiotics on Graphene and Biochar in Aqueous Solutions Induced by π-π Interactions

**DOI:** 10.1038/srep31920

**Published:** 2016-08-18

**Authors:** Bingquan Peng, Liang Chen, Chenjing Que, Ke Yang, Fei Deng, Xiaoyong Deng, Guosheng Shi, Gang Xu, Minghong Wu

**Affiliations:** 1 School of Environmental and Chemical Engineering, Shanghai University, 99 Shangda Road, Shanghai 200444, China; 2 Division of Interfacial Water and Key Laboratory of Interfacial Physics and Technology, Shanghai Institute of Applied Physics, Chinese Academy of Sciences, Shanghai 201800, China; 3 School of Science, Zhejiang Agriculture and Forestry University, Lin’an, Zhejiang 311300, China; 4 Shanghai Applied Radiation Institute, Shanghai University, 99 Shangda Road, Shanghai 200444, China

## Abstract

The use of carbon based materials on the removal of antibiotics with high concentrations has been well studied, however the effect of this removal method is not clear on the actual concentration of environments, such as the hospital wastewater, sewage treatment plants and aquaculture wastewater. In this study, experimental studies on the adsorption of 7 antibiotics in environmental concentration of aqueous solutions by carbon based materials have been observed. Three kinds of carbon materials have shown very fast adsorption to antibiotics by liquid chromatography–tandem mass spectrometry (LC-MS-MS) detection, and the highest removal efficiency of antibiotics could reach to 100% within the range of detection limit. Surprisedly, the adsorption rate of graphene with small specific surface area was stronger than other two biochar, and adsorption rate of the two biochar which have approximate specific surface and different carbonization degree, was significantly different. The key point to the present observation were the π-π interactions between aromatic rings on adsorbed substance and carbon based materials by confocal laser scanning microscope observation. Moreover, adsorption energy markedly increased with increasing number of the π rings by using the density functional theory (DFT), showing the particular importance of π-π interactions in the adsorption process.

Antibiotics have been used for several decades in both human and animals for treatment of microbial infections, and also as feed additives for promotion of growth of livestock animals^1^,^2^,^3^. Antibiotics of global production^4^,^5^ eventually enters into the environment after usage and as residuals in sewage. High concentrations of antibiotics have been detected in hospital effluent (124.5 ng/ml for Ciprofloxacin^6^, wastewater treatment plant influents (64 ng/ml for cephalexin^7^, 80 ng/ml for β-lactams^8^,^9^ and culture wastewater (540 ng/ml for Tetracyclines, 275 ng/ml for Macrolides^10^. The sewage of hospitals, pharmaceutical factories and sewage treatment plants was an important repository of the resistance genes^11^,^12^, which need to be effectively treated before discharging into the natural water body. Unfortunately, the removal of antibiotics by conventional technologies in wastewater treatment plants, such as, β-Lactams (17–43%)^13^, Macrolides (40–46%)^14^, Sulfonamides (20–24%)^15^, Tetracyclines (66–90%)^16^ were generally incomplete^17^. The techniques biological treatments, membrane filtration, activated carbon adsorption, advanced oxidation processes (AOPs), and disinfection on different classes of antibiotics have been widely investigated^18^. It is estimated that only part of antibiotics can be degraded during biological treatments^19^ or UV disinfection^20^ of water and wastewater. Both AOPs and membrane filtration have a high removal efficiency (90–99%^21^ and 98.5%^22^ of antibiotics, but the methods are restricted by high cost and harsh conditions. Adsorption is considered very effective method for removal of antibiotics with high concentrations (from a few to several hundred ug/ml) in water or wastewater^5^,^23^,^24^,^25^,^26^,^27^,^28^,^29^. However, the concentrations of the experiments were much higher than that of the environments. There were few reports on the adsorption of environmental antibiotics at the concentrations of <1 ug/ml on carbon based materials. Except for the problem of production of large amount of antibiotic resistant genes^11^,^12^, the presence of the detected concentration antibiotics in wastewater might disturb the stability and performance of microbial communities in environments due to the strong bacteriostatic effects of antibiotics. Therefore, there is still an increasing demand for the development of efficient and cost-effective carbon-based material adsorption processes for the removal of antibiotics.

Carbon based materials are widely used in biomedicine^30^, environmental detection (solid phase microextraction)^31^, adsorption of organic compounds^32^, wastewater treatment^33^, indoor air purification^34^, potable water purification^35^ and soil amendment in agricultural and environmental applications^36^. Since they have a high adsorption capacity and removal efficiency for certain organic compounds, they are widely used to remove organic contaminants from industrial water. Current research indicates that there are multiple mechanisms of action, for example, hydrophobic effect, π-π interactions, hydrogen bonds, covalent and electrostatic interactions. These mechanisms coexist in the adsorption process of organic pollutants by carbon-based materials^32^. However, the main adsorption mechanism of carbon based materials is still not clear.

In this paper, seven commonly used antibiotics with near actual environmental concentrations were taken as examples, and two kinds of biochar with large surface area and a graphene with small surface area were chosen as sorbents. The objectives of this study were: (1) to determine the adsorption ability of biochar or graphene on antibiotics in environmental concentration, and draw support from fluorescence experiments to directly observe the adsorption process, (2) to reveal the effects of antibiotics with different aromatic rings on adsorption, (3) to explain the adsorption mechanism by experiment and density functional theory. The concentration of antibiotics in the samples were detected by liquid chromatography–tandem mass spectrometry (LC-MS-MS). We found that three sorbents showed rapid adsorption to antibiotics. The graphene with small specific surface area had the best adsorption capacity. For the same sorbents, the number of aromatic rings on antibiotics is a main factor affecting the adsorption rate, which is in agreement with the results of density functional calculations. The main adsorption mechanism is π-π interactions deduced by observing the adsorption process in confocal laser scanning microscope from fluorescence experiments. The cheap carbon materials with more aromatic ring area (e.g., bamboo biochar prepared by high temperature) can rapidly and efficiently remove the antibiotics from the actual environment. It is also expected to have great applications in removal of polycyclic aromatic hydrocarbons in water.

## Results and Discussion

### Characterization of Graphene and Biochars

**Figure 1:**
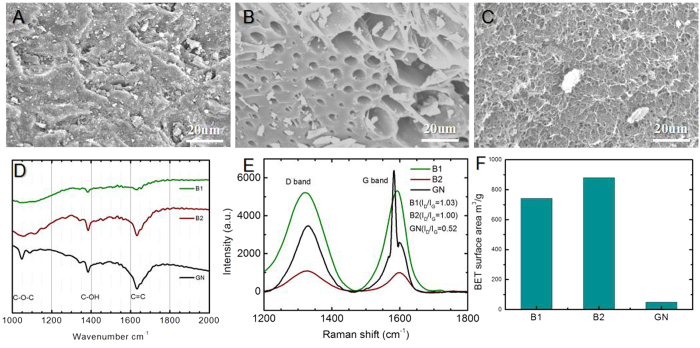
Characterization of B1, B2, and GN. (**A–C**) SEM images; (**D**) FTIR; (**E**) Raman shift; (**F**) surface area of B1, B2, GN.

The morphologies of graphene and biochar were visualized by using scanning electron microscope (SEM). As shown in Fig. 1A–C, the microstructure among three kinds of carbon materials is distinguishing. Micron level multi-gap structures were not observed on B1. This might be the result of different production processes. B2 surface is a porous structure. Wrinkled and non porous structures were observed on the surface of GN. As shown in the Fourier transform infrared spectroscopy (FTIR) spectra in Fig. 1D, the graphene and biochar presented the various functional groups: absorption peaks characteristics of each sample were substantially the same, indicating that they have the same type of groups on surface. The functional groups -OH (at approximately 3450 cm^−1^), C=C (at approximately 1630 cm^−1^), C-OH (at approximately 1400 cm^−1^), and C-O-C groups (at approximately1050 cm^−1^) were observed on the surface for graphene and biochar^37^,^38^. Three samples all have absorption peaks in the wave numbers, 3450 cm^−1^, 1630 cm^−1^, 1400 cm^−1^ and 1050 cm^−1^, showing the surface contained carboxyl, phenolic hydroxyl and oxygen containing groups. The broad peak at approximately 3450 cm^−1^ was assigned as stretching vibration of adsorbed water, and there was not a distinction among three carbon materials. But the absorption peaks of GN and B2 at 1630 cm^−1^ were stronger than that of B1, which means that the content of aromatic ring on the surface of GN and B2 was higher than on B1. This C-O-C (1050 cm^−1^) functional group, suggested that oxygen-containing groups were introduced into the graphene.

Raman spectra of the B1, B2 and graphene can be found in Fig. 1E. The Raman spectrum of the graphene and biochars consists of two signature bands: a sharp G band at 1560–1600 cm^−1^, and a D band at 1320–1350 cm^−1^. The D band relates to disordered sp^2^-hybridized carbon atoms containing vacancies, impurities, or other symmetry-breaking defects, such as oxygen containing groups, whereas the G band represents the structural integrity of sp^2^-hybridized carbon atoms. The extent of carbon-containing defects in the biochars and graphene can be estimated by an intensity ratio of D band to G band (I_D_/I_G_)^39^. The I_D_/I_G_ ratios for the B1, B2 and graphene are 1.03, 1.00 and 0.52, respectively. The higher I_D_/I_G_ ratio in biochars suggests that biochars possessed fewer aromatic rings structures and more carbon-containing defects that led to the formation of oxygen-containing functional groups on the surface of biochars^40^. In addition, according to analysis of the elements and functional groups of graphene by SEM, graphene was relatively pure, containing only C and O elements (hydrogen was not be detected), and the atomic percentages were 98.17% and 1.83%, respectively. The Brunauer-Emmett-Teller (BET) surface area was measured by standard BET equation applied in the relative pressure range of 0.05–0.3 as shown in Fig. 1F. The BET surface area of B1, B2, GN were 742.1679 m^2^/g, 881.6748 m^2^/g and 49.3840 m^2^/g, respectively. The surface area of biochar was obviously higher than that of graphene.

### Determinations of the Adsorption Equilibrium Time

**Figure 2:**
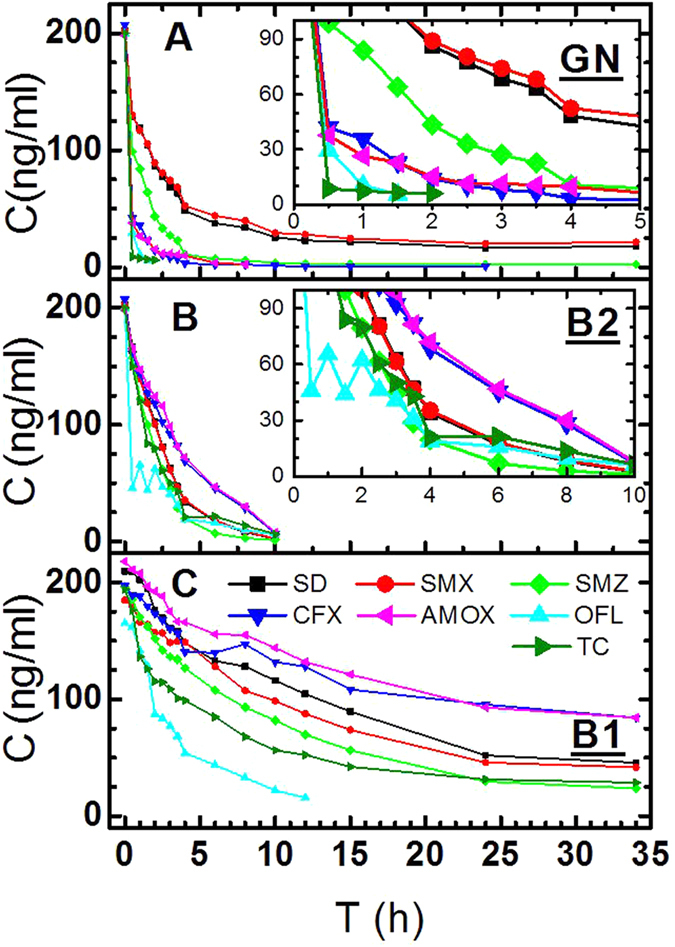
The change of the concentration of antibiotics in solution with time for 3 adsorbents. grapheme (GN); bamboo biochar (B2); coconut shell biochar (B1).

The adsorption processes of various antibiotics on carbons is shown in Fig. 2. The data that are lower than the detection limit is considered as invalid data. Graphene and two biochar have strong adsorption efficiency on seven antibiotics. At the same time, the blank experiment without carbon based materials showed that average concentrations of the seven antibiotics in 34 hours were 200.12 ng/ml (SD), 208.86 ng/ml (SMXZ), 201.03 ng/ml (SMZ), 188.68 ng/ml (CFX), 168.97 ng/ml (OFL), 196.97 ng/ml (AMOX) and 194.3396 ng/ml (TC), respectively. The concentrations of the blank samples were close to the original concentration of 200 ng/ml, which were indicative of the stabilization of the blank samples. The results could eliminate the other interference in the process of the experiment, and the added carbon based materials were the only factor of changes in the concentration of the sample. The adsorption equilibrium time was determined by the antibiotic concentration changes with time. With the extension of the adsorption time, the adsorption rate had no obvious change, the adsorption reached balance or complete absorption because of extremely low concentration of antibiotics. In addition, the three kinds of adsorbents showed different adsorption behavior, and adsorption response of the seven kinds of antibiotic on same adsorbent were also different. For B1, adsorption equilibrium time of OFL was about 15 h and shorter than that of other six kinds of antibiotics, which were about 30 h; and the concentration of OFL and TC were falling fast within 5 h; then it slowly tended to be stable. Other antibiotics showed relatively slow adsorption. B2 adsorption effect of 7 antibiotics was more obvious than B1, and it has reached equilibrium after 10 h (lower than detection limits 0.48–4.8 ng/ml), especially for the concentration of OFL that sharply declined in 2 h, and then slowly leveled off. For GN, the fastest adsorption rate of antibiotics was presented in 2 h. The adsorption equilibrium times in graphene for each antibiotic were about 2.5 h for TC and OFL; 12 h for CFX, 15 h for AMOX, AMZ and SD; 24 h for SMX. From Fig. 2, experimental results showed that these antibiotics in environmental concentrations can be almost completely removed by GN and B2 (89.3–100% and 100% of removal efficiency) when reaching the adsorption equilibrium, here, we decided that antibiotics were complete removal when the concentration of the antibiotics were lower than the detection limit. Though the B1 did not remove all antibiotics after 34 hours under the same condition (57.9–100% of removal efficiency), it was found that all antibiotics can be removed by increasing the appropriate amount after 30 hours.

### Adsorption Kinetics Simulation

**Figure 3:**
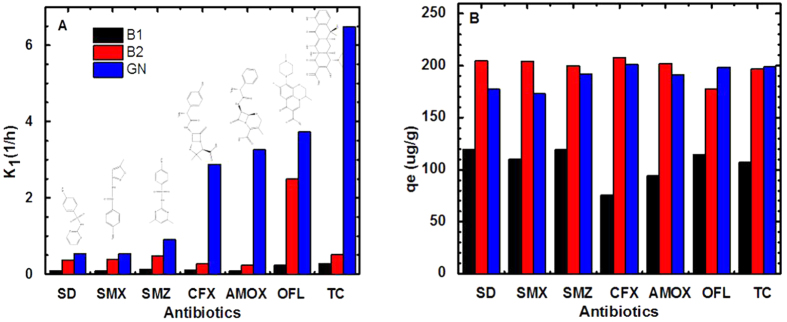
Adsorption kinetics equations of first order. (**A**) k_1_ (1/h) rate constant of the first-order kinetic model; (**B**) q_e_ (ug/g) is the mass of antibiotics adsorbed on per unit mass of adsorbent at equilibrium time.

The adsorption test is the study on the adsorption of trace antibiotics of different carbon based materials, thus some antibiotics reached the removal in the adsorption balance. More concern was given to the adsorption rate of carbon based materials to antibiotics. In order to qualificatory compare the adsorption rate of biochar and graphene with 7 kinds of antibiotics, the adsorption kinetics model was used to simulate the experimental data. Adsorption kinetics is commonly interpreted by first- and second-order kinetic models. When the concentration of the antibiotic was lower than the detection limit, we decided it was 0 ng/ml. According to equations^41^,^42^ (pseudo-first-order kinetic model (Equation1) and pseudo-second-order kinetic model (Equation 2)) experimental data is fitted by using Origin.
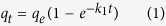

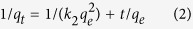
where q_e_ and q_t_ (ug/g) is the mass of antibiotics adsorbed on per unit mass of adsorbent at equilibrium and at time t (h), respectively. k_1_ (1/h) is rate constant of the first-order kinetic model, k_2_ (g/ug/h) is rate constant of the second-order kinetic model. Adsorption data of biochar and graphene adsorbed antibiotic are modeled by the above two kinetic models, and the results are shown in Table 2S. Comparison of fitting results of Table 2S, the correlation coefficients (R^2^) of pseudo-first-order kinetic and pseudo-second-order kinetic model are almost the same, however the theoretical values of equilibrium adsorption amount (q_e_), calculated from the first-order kinetics models were in good agreement with the experimental values. According to the adsorption rate parameter (K_1_) of first order adsorption kinetics equations, as shown in Fig. 3, for three kinds of carbon materials for adsorption of antibiotics, the speeds from fast to slow follow the order: GN > B2 > B1. The adsorption rate of the same carbon materials for 7 kinds of antibiotics follows this order: (B1) TC > OFL > SMZ > CFX > AMOX > SD > SMX; (B2) OFL > TC > SMZ > SMX > SD > CFX > AMOX; (GN) TC > OFL > AMOX > CFX > SMZ > SMX > SD. For the same adsorbent, the different adsorption rates of antibiotics were related to the structures of the antibiotics. Detailed analysis is discussed in the mechanism analysis section. We note that the concentration of SD and SMX adsorb by GN decreased faster than that of B2 at first 2 hours, however, the speed of decrease are reversed in the next time. According to equation (1), the curves of the concentration of antibiotics (in Fig. 2) are determined by the q_e_ and k_1_ parameters. For SD and SMX, the q_e_ of B2 is larger than that of GN since the BET surface area of B2 is significant higher than that of GN which means larger adsorption capacity of B2. Thus, the smaller BET surface ar_e_a of GN affect the decreasing of concentration after 2 hours.

### Adsorption capacity

The researches of carbon based materials for adsorption of antibiotics confirmed that carbon based materials had a strong adsorption capacity^5^,^23^,^24^,^25^,^26^,^27^,^28^,^29^,^43^. For the same amounts of adsorbate, the adsorption capacity of GN and B2 were relatively close, which was stronger than that of B1, as shown in Fig. 3B. The mean values of q_e_ for B1, B2, GN were 105.7, 199.0, 190.3 ug/g respectively.

Research^22^ showed that the adsorption capacity of GN to TC was stronger than to biochar, and adsorption of bulky TC was much lower on the activated carbons than low sized SMX due to the size-exclusion effect. However, in this study as shown in Fig. 2, the adsorption of bulky TC was stronger than that of low sized SMX on the biochar and graphene, which indicated that carbon based materials adsorption of antibiotics at environmental concentrations was without size-exclusion effect. The adsorption capacity of porous B2 and nonporous GN was very strong, B1 was relatively weak, and the specific surface area of B1 and B2 was bigger than for GN. Thus, the porosity and the surface area were not major factors in determining the adsorption efficiency. In comparison with the results of our carbon materials characterization and adsorption experiments, it was found that the functional groups C=C of both carbon based materials and antibiotics was an important factor in determining the adsorption rate, the more aromatic ring is, the faster the adsorption rate will be.

It is worth noting that the concentration of antibiotics has an important influence on the adsorption process. The adsorption equilibrium time of activated carbon for high concentration AMOX (317 mg/L) is only 35 min, and the values of q_e_ can reach 25 mg/g^29^. The adsorption capacity increases with the increase of concentration. Carbon based materials exhibits excellent adsorption properties that have great adsorption capacity for removal of antibiotics not only in low environmental concentrations, but also in high concentrations.

### Visualisation of Carbon-Based Material Adsorption of Fluorescein Isothiocyanate (FITC)

**Figure 4:**
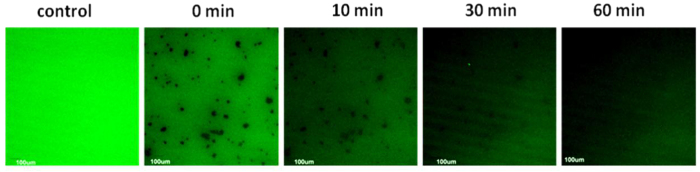
Fluorescence image of graphene adsorption of FITC at 0, 10, 30, 60 (min).

It was observed that graphene had an obvious adsorption effect on FITC in one hour. The fluorescence images at different times (0, 10, 30, 60 min) were observed under the same conditions in the same field. The fluorescence results of graphene adsorption of FITC with time were shown in Fig. 4. With the increasing of adsorption time, the brightness of the fluorescence was significantly decreased in solution. There was a marked difference between the control and the 0 min of the fluorescence intensity, because of about 2 minutes preparation time before the fluorescence observation. FITC had been adsorbed fastly during the preparation time. The addition of graphene particles resulted in fast fluorescence quenching of FITC solution. As for the adsorption mechanism in this system it could occur by chemisorption, hydrogen bonding interaction, electrostatic interaction and π-π interactions. It is well known that FITC can bind to various proteins^44^, mainly through amino-groups in protein and thiocarbamide of fluorescein forming chemical bonds, and their combination still has a strong yellow green fluorescence in solution. It has no obvious evidence for the involvement of hydrogen bonds in fluorescence quenching^45^. If hydrogen bonds or electrostatic force are formed, the adsorption sites should be charged amino and thiocarbamide groups of FITC, which are similar in structure to the chemisorption. The π-π overlap of physisorption between the FITC and graphene may involves a energy transfer or electron transfer interaction^46^ between the FITC and graphene, π-π interactions in a stacked conformation resulted in very efficient fluorescence quenching^47^,^48^. Thus, the adsorption of chemisorption and hydrogen bonding interaction will not reduce the fluorescence intensity of FITC, in contrast, FITC should be condensed on adsorbents and enhances fluorescence. So it can be inferred that the fast fluorescence quenching of FITC is driven by adsorption of graphene through π-π interactions.

### Adsorption Mechanism

As previously reported, the adsorption behavior of organic compounds on carbon based material in general follows mechanisms such as π-π interaction^49^,^50^, hydrophobic interaction, H-bonding interaction^51^, electrostatic interaction^48^,^52^, pore-filling mechanism^53^, or the simultaneous occurrence of several adsorption mechanisms^54^. The graphene in this study was pure and had small specific surface area with few pores and functional groups, but its ability to adsorb antibiotics was the strongest. Although B1 and B2 have similar specific surface area and type of groups on surface, the effect of adsorption antibiotics on B2 is stronger, due to more aromatic ring area on B2 than B1 by different degree of high-temperature activation (1000 °C and 800 °C). It is in good agreement with the report that adsorption depends on the carbonization degree of biochars and the concentration of adsorbate^55^. From the view of antibiotics, the number of aromatic rings on antibiotics was also an important factor affecting the adsorption rate. Here, we define hexagonal ring molecular structure as π-ring. It is concluded that the more aromatic rings the antibiotics have, the faster is the adsorption rate on the carbon-based materials. The number of π-ring on seven kinds of antibiotics follows the order: TC (4) = OFL (4) > AMOX (2) = SMZ (2) = SD (2) > CFX (1) = SMX (1), which is roughly consistent with the order of reaction kinetics simulation rate. According to the reaction rate parameter (K_1_) as shown in Fig. 3A, the adsorption of TC and OFL are the fastest, while SD and SMX are the slowest. In addition, we have used density functional theory (DFT) simulations to find the result that the interaction between π rings and graphene were sufficiently strong, and the adsorption energies increased with number of the π rings. The details are presented in the following computational study. According to the above adsorption experimental data and analysis, it indicates that the adsorption is determined mainly by the number or areas of aromatic rings both in antibiotics and adsorbent, the main adsorption mechanism is the π-π interaction. The conclusions are also supported by the experiment of the fast fluorescence quenching of FITC by adsorption of graphene. The origin of the hydrophobic effect is not fully understood, it can be a comprehensive expression of hydrogen bond and π-π interaction. And the functional groups on the antibiotics, pore-filling of porous biochar may has a minor effect on the adsorption behavior.

### Adsorption of π Rings on the Graphite Flake Surface by Density functional theory

**Figure 5:**
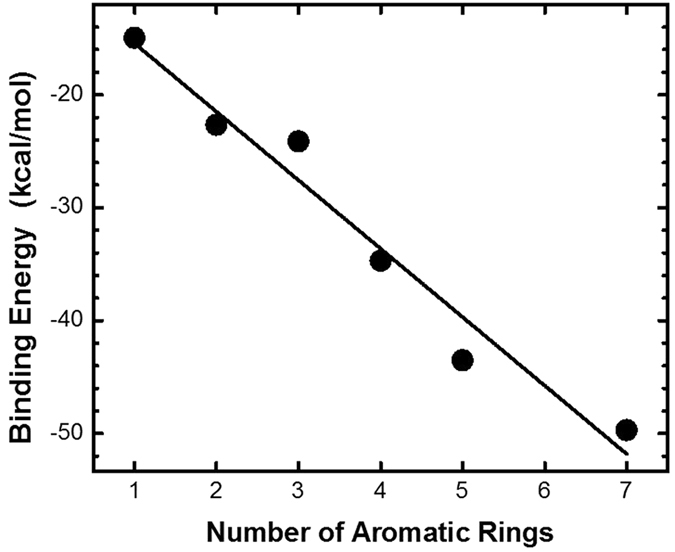
Adsorption energy of the different π rings on graphene flake surface.

In order to verify the adsorption mechanism of π rings on graphene and biochar, and the adsorption correlation between graphene and π rings with various number, here, we analyzed the adsorption of a series of increased size π rings on a graphene flake at the ωB97X-D/6-31 + G(d,p) levels of theory. The optioned structures are showed in Figure S1. The adsorption energies of various sized π rings are shown in Fig. 5. The smallest value was −14.97 kcal/mol, where π-π interaction was equivalent to ~25 kBT for T = 300 K (about triple of the value of hydrogen-bond energy between two water molecules)^56^, indicating that π ring adsorption on this graphene flake is quite stable at room temperature. The adsorption energies increase from −14.97 to −49.69 kcal/mol with increasing size of the π ring. The interaction between π rings and graphene flake are sufficiently strong to result in partial dehydration of the π rings, i.e., benzene will displace some water molecules from direct contact with the ion^57^. The positive correlation between the adsorption energy and the number of aromatic rings is consistent with the experimental results.

## Conclusions

Carbon based materials are increasingly recognized as effective, inexpensive, and environmentally friendly sorbents for abating organic contaminants. In this paper, the adsorption of 7 antibiotics by 2 biochar and graphene were studied in actual concentration of environments such as the hospital wastewater, sewage treatment plants and aquaculture wastewater, which pose antibiotics resistance genes, potential human health and ecological risks. In the three cabon based materials, the B1 has large surface area and less aromatic ring area by 800 °C carbonization degree; the B2 has porous, large surface area, high aromatic ring area by 1000 °C carbonization degree; and the GN has high aromatic ring areas, and small specific surface area with few pores and functional groups. The adsorption kinetics model was used to simulate the experimental data. And adsorption processes of FITC, which has similar groups and molecular weight with antibiotics, by graphene particles were visualized by fluorescence microscope.

The results show that the three carbon based materials have good adsorption proporties for antibiotics in actual concentration of environments, by which the highest removal efficiency of antibiotics can be up to 100%. The adsorption ability follows this order: GN > B2 > B1. From the view of antibiotics adsorption on same adsorbents, the number of aromatic rings on antibiotics was also an important factor in determining the adsorption rate, the more aromatic ring is, the faster the adsorption rate will be. The adsorption process by graphene lead to fast fluorescence quenching of FITC in 1 hour via π-π interaction. We have used density functional theory simulations to find the strong interaction between π rings and graphene, and the adsorption energies increased with number of the π rings. It is concluded that the main adsorption mechanism is the π-π interaction, and it also should be mentioned that other adsorption mechanism such as hydrophobic interaction, H-bonding interaction, electrostatic interaction, pore-filling could not be excluded, which may be auxiliary adsorption mechanism.

Comparing the sorption of antibiotics by 3 kind carbon based materials, the biochar with high carbonization degree or graphene, which has rich aromatic ring areas, were more effective in adsorption of antibiotics. From the view of adsorption mechanism, antibiotics with more aromatic rings should be recommended in applications for the easy removal of antibiotics in the environment. At the same time, an efficient, fast and simple sewage treatment process is needed to remove the antibiotics from hospital wastewater, pharmaceutical wastewater and sewage treatment plants.

## Materials and Methods

### Antibiotics and reagents

**Figure 6:**
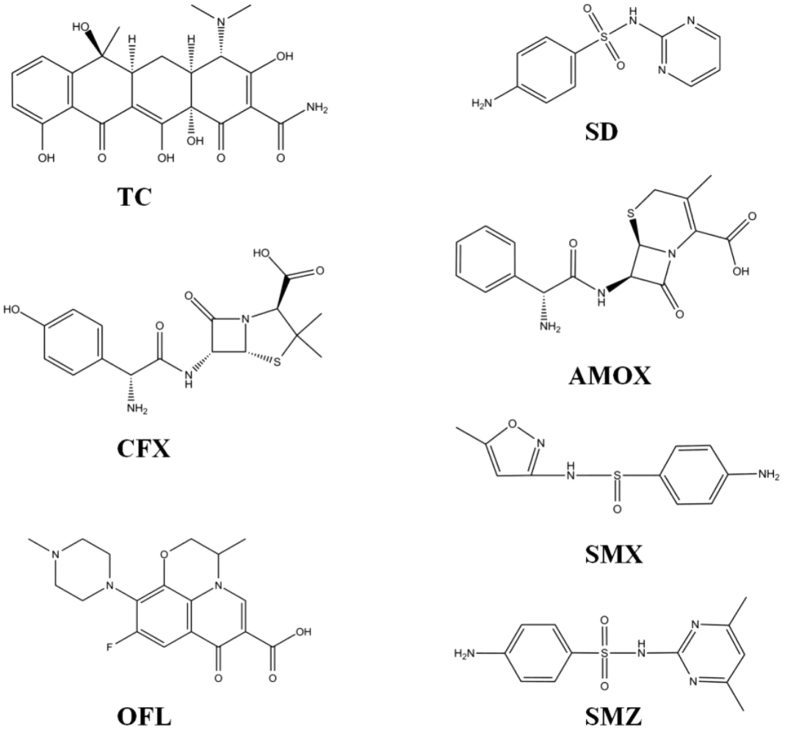
Chemical structure of antibiotics.

Seven antibiotic standards (one fluoroquinolon, three sulfonamides, two β-Lactams and one tetracycline) including Ofloxacin (OFL), Sulfadiazine (SD), Sulfamethoxazole (SMX), Sulfamethazine (SMZ), Cefalexin (CFX), Amoxicillin (AMOX), Tetracycline (TC) were purchased from Dr. Ehrenstofer GmbH (Germany). Chemical structures are shown in Fig. 6. HPLC grade methanol and acetonitrile were purchased from Merck(Germany). Ultra-pure water was produced by a Milli-Q water purification system.

### Adsorbents

Two types of commercial biochars (Coconut shell biochar, Bamboo biochar) and one kind of homemade graphene^58^ were used. Coconut shell carbon, bamboo biochar, and graphene were denoted as B1, B2 and GN, respectively. Analytics project test data: B1: Iodine value or 1100 mg/g, 800 °C high-temperature activation, pH value 7–9; B2: Iodine value 1420 mg/g, 1000 °C high-temperature activation, pH value 7–9.

In order to keep the biochar particles size uniformity, eliminate the interference of other substances and microbial interference in biochars, the original samples were treated as follows: the particle size of the biochar was 0.4–0.8 mm after grinding. The biochars and graphene samples were washed by deionized water to an invariable pH value and desiccated by vacuum freeze–drying. The microbial interference was excluded by 30 min UV irradiation. Finally, the carbon materials were characterized by SEM, FTIR spectrum and Raman spectrometer (InVia plus, Renishaw plc, Britain). Surface area of adsorbents (biochar and garphene) were determined by nitrogen adsorption using ASAP 2020 V3.04 H (Micromeritics).

### Experimental design

The concentrations of antibiotics in the sewage of hospitals, pharmaceutical plants and sewage treatment plants have been reported in the range of from 64 ng/ml to 540 ng/ml^6^,^7^,^8^,^9^,^10^. In order to simulate the adsorption of antibiotics in actual water environment, the 200 ng/ml concentration of antibiotics (C_0_) was chosen as initial concentration.

Static adsorption method^41^,^42^ was used to study the adsorption process and effect of biochars and graphene. For the kinetic studies, 100 mg of adsorbent was put into 100 ml of antibiotics solution which contained seven antibiotics, 200 ng/ml per antibiotic, pH 7.0. The adsorption was carried out at 298 K, with the oscillation rate 120 r/min and intermittent sampling after the solids were separated by using a 0.22-μm Hydrophilic PTFE syringe filters (SCAA-114, Anpel), filtrated for the determination of antibiotic group concentration the adsorption equilibrium time. The change of adsorption amount (q) with time (t) in the process of the adsorption process and adsorption kinetics analysis of biochar and graphene were obtained. Each batch of test set blank interference experiment excluded degradation.

### Fluorescence Observation

In order to visually observe the carbon-based material adsorbed antibiotic, the fluorescence properties of the 7 kinds of antibiotics were detected, the results showed that the fluorescence brightness was not suitable for direct observation of the adsorption process. The fluorescent agent fluorescein isothiocyanate (FITC), whose structure is similar to antibiotics (containing aromatic rings, functional groups) was chosen to directly observe the adsorption process. The molecular weight of FITC is 389.4, which is in the range of antibiotics (251.2-445.2) as listed in Table S1. The adsorption process of fluorescent agent by carbon-based material could lead to change of fluorescence brightness and distribution in solution. Graphene was chosen to adsorb FITC, by using confocal laser scanning microscope to observe the change in fluorescence over time.

The suitable amount of graphene particles was added to the solution of FITC with concentration of 0.1 mg/ml, and then the sample was placed on the stage. By using visual mode, the magnification of the objective was adjusted; the need to test the samples under the fluorescence microscope was found (FLUOVIEW FV1000). By switching to a scanning mode, the double needle and laser intensity parameters, could get clear confocal images. Under the same parameters, we observed the fluorescence change in the same field within one hour.

## Additional Information

**How to cite this article**: Peng, B. *et al*. Adsorption of Antibiotics on Graphene and Biochar in Aqueous Solutions Induced by π-π Interactions. *Sci. Rep.* **6**, 31920; doi: 10.1038/srep31920 (2016).

## Supplementary Material

Supplementary Information
