# Supplementary material for: Adsorption of Antibiotics on Graphene and Biochar in Aqueous Solutions Induced by π-π Interactions
Source: Sci Rep. 2016 Aug 18;6:31920. doi: 10.1038/srep31920 (PMC4989150; doi:10.1038/srep31920)
Supplement: Supplementary Information [file srep31920-s1.doc]

**Supplementary Material For**

**Adsorption of Antibiotics on Graphene and Biochar in Aqueous Solutions Induced by π–π Interactions**

Bingquan Peng^1,+^, Liang Chen^1,2,4,+^, Chenjing Que^1^, Ke, Yang^1^, Fei Deng^1^, Xiaoyong Deng^1^, Guosheng Shi^2,*^, Gang Xu^1,*^, Minghong Wu^3,*^

^1^School of Environmental and Chemical Engineering, Shanghai University, 99 Shangda Road, Shanghai 200444, China

^2^Division of Interfacial Water and Key Laboratory of Interfacial Physics and Technology, Shanghai Institute of Applied Physics, Chinese Academy of Sciences, Shanghai 201800, China

^3^Shanghai Applied Radiation Institute, Shanghai University, 99 Shangda Road, Shanghai 200444, China

^4^School of Science, Zhejiang Agriculture and Forestry University, Lin'an, Zhejiang 311300, China

**Methods and Density functional theory**

**Tables S1 and S2**

**Figure S1 and S2**

**Reference List**

**1 Methods**

Analysis of antibiotics by LC-MS-MS. The analysis of all target antibiotics were performed on an Agilent 1260 liquid chromatograph coupled to an Agilent 6460 triple quadrupole mass spectrometer. A Poroshell 120 EC-C18 reversed-phase column (3×100 mm, 2.7 μm, Agilent) was used for the determination of antibiotics and the column temperature was set to 40 °C. Calibration curves were used for identification and quantification of target antibiotics (7 calibration standards ranging in concentration from 2 to 200 ng/ml for all targets)

Sample injection volume was 5 μL and the flow rate was 0.4 ml/min. The mobile phases used for the analysis was acetonitrile as eluent A and water with 0.2% (v/v) formic acid and 5 mmol/L ammonium acetate as eluent B. The elution gradient program was set up as follows: 0-5 min, 0% -13%A; 5-10 min, 13%-20%A; 10-32 min, 20%-90%A, and post time is 5 min. The instrument conditions were as follow: capillary voltage, 4.5 kV; drying gas temperature, 350 °C; source gas flow, 10 L/min; and drying gas 3 L/min. The optimized ESI-MS/MS conditions for analysis of target antibiotics are summarized in Table S1.

**Table S1．** Instrumental analysis data of 7 antibiotics

|  | retention time  (min) | precursor  ion  (m/z) | quantitative ion  (m/z) | Qualitativeion  (m/z) | Fragment  (V) | [collision](javascript:void(0);) energy (eV) | Cone voltage  (eV) | detection limit  (ng/L)  (S/N=10) |
| --- | --- | --- | --- | --- | --- | --- | --- | --- |
| SD | 3.28 | 251.2 | 156.0 | 108.1 | 114 | 14 | 21 | 1.8670 |
| SMX | 10.83 | 254.1 | 156.1 | 92.1 | 118 | 14 | 34 | 0.7620 |
| SMZ | 6.95 | 279.1 | 124.1 | 92.1 | 118 | 30 | 26 | 0.4830 |
| CFX | 2.86 | 348.2 | 158.0 | 106.0 | 82 | 5 | 29 | 4.8000 |
| AMOX | 1.36 | 366.1 | 349.0 | 113.9 | 91 | 5 | 17 | 2.5600 |
| OFL | 7.14 | 362.1 | 261.2 | 443.2 | 100 | 28 | 10 | 1.7140 |
| TC | 8.36 | 445.2 | 410.1 | 427.2 | 132 | 18 | 20 | 2.3680 |

**2 Density functional theory**

The dispersion-corrected DFT functional ωB97X-D^1^ was used to geometrically optimize the structures of the various π–π complexes. The electron wave functions in the Gaussian function basis were used. For geometry optimization, the double-ζ basis^2^ was used. At the same time, we introduced a diffuse function into the basis set and added a d-polarization function to the carbon atoms and ions, as well as a p-polarization function to the hydrogen atoms (6-31+G(d,p)). The geometry optimizations were performed using the Berny algorithm^3^ with the convergence criteria of maximum step size of 0.0018 au and RMS force of 0.0003 au. ωB97X-D calculations were carried out using the Gaussian 09^4^ package. The geometry optimizations of all the complexes were performed without any symmetry constraints for all the computational methods. The adsorption energies (Δ*E*_ads_) of π rings to the graphene flake were defined as:

$$\Delta E_{\text{ads}}=E_{\pi-f}-E_{\pi}-E_{flake}$$

where *E*_π_, *E*_flake_, and *E*_π-f_ are the total energies of the π ring, graphene flake and the π rings complex, respectively.

**Table S2.** Adsorption kinetics equations of first order and pseudo second order

| Sample | | q_e_(exp)^a^  (ug/g) | First-order model | | |  | Second-order model | | |
| --- | --- | --- | --- | --- | --- | --- | --- | --- | --- |
|  |  |  | K_1_  (1/h) | q_e_(cal)^b^ (ug/g) | R^2^ |  | K_2_ (g/(ug·h)) | qe(cal)^b^ (ug/g) | R^2^ |
| **B1** | SD | 114.7984 | 0.08952 | 119.5685 | 0.98234 |  | 0.000468 | 161.6150 | 0.98583 |
|  | SMX | 100.1704 | 0.08074 | 109.7695 | 0.99322 |  | 0.000415 | 153.6464 | 0.99039 |
|  | SMZ | 120.7621 | 0.12577 | 119.1750 | 0.99003 |  | 0.000783 | 152.1282 | 0.99723 |
|  | CFX | 78.9817 | 0.11051 | 76.07325 | 0.94675 |  | 0.001060 | 97.8656 | 0.96056 |
|  | OFL | 115.5862 | 0.24477 | 114.2000 | 0.97568 |  | 0.001910 | 136.9864 | 0.96731 |
|  | AMOX | 93.5964 | 0.09626 | 94.0376 | 0.97187 |  | 0.000694 | 123.8968 | 0.98120 |
|  | TC | 115.8652 | 0.28265 | 106.9280 | 0.95225 |  | 0.002682 | 124.6949 | 0.98284 |
| **B2** | SD | 202.5753 | 0.37602 | 204.6741 | 0.99601 |  | 0.001989 | 233.6973 | 0.97176 |
|  | SMX | 202.5866 | 0.38186 | 204.2262 | 0.99691 |  | 0.002044 | 232.7600 | 0.97451 |
|  | SMZ | 198.4026 | 0.48209 | 200.0174 | 0.99681 |  | 0.002887 | 223.6500 | 0.96926 |
|  | CFX | 207.1777 | 0.28334 | 207.6613 | 0.98673 |  | 0.001400 | 241.0369 | 0.98342 |
|  | OFL | 199.2470 | 2.50212 | 177.9172 | 0.79729 |  | 0.015976 | 192.7417 | 0.89866 |
|  | AMOX | 198.1897 | 0.23950 | 202.2324 | 0.99283 |  | 0.001107 | 239.9746 | 0.97821 |
|  | TC | 200.2202 | 0.51093 | 196.6060 | 0.99030 |  | 0.003203 | 218.9929 | 0.98601 |
| **GN** | SD | 184.8542 | 0.53594 | 177.5054 | 0.95853 |  | 0.004015 | 195.6149 | 0.98524 |
|  | SMX | 181.2760 | 0.53933 | 172.8584 | 0.94906 |  | 0.004175 | 190.3892 | 0.98448 |
|  | SMZ | 196.2613 | 0.90569 | 191.9216 | 0.96856 |  | 0.007515 | 206.0007 | 0.98713 |
|  | CFX | 207.1777 | 2.88418 | 201.2164 | 0.97385 |  | 0.030458 | 208.6676 | 0.99560 |
|  | OFL | 199.2470 | 3.72796 | 198.6203 | 0.99846 |  | 0.060664 | 202.3590 | 0.99742 |
|  | AMOX | 198.1897 | 3.26616 | 191.1386 | 0.97540 |  | 0.036339 | 197.8739 | 0.99625 |
|  | TC | 200.2202 | 6.48594 | 198.8393 | 0.99701 |  | 0.169959 | 200.6055 | 0.99882 |

^a^ Experimental data.

^b^ Calculated data from models.


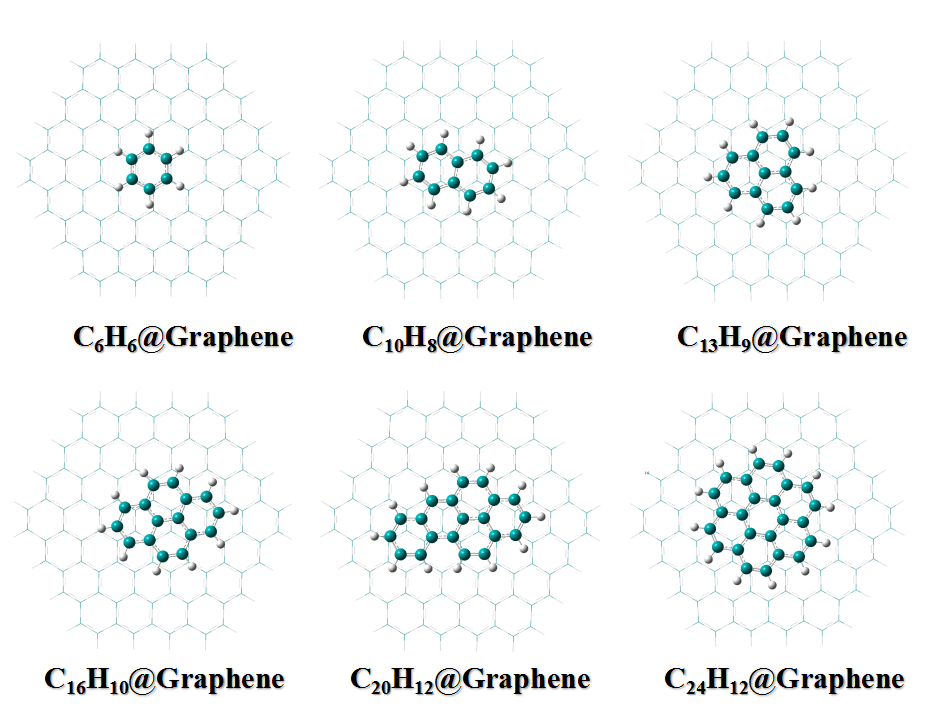


**Figure S1.** Stable structures of the different-sized π rings adsorption to the graphene flake at the ωB97X-D/6-31+G(d,p) level of theory. Gray and white balls represent carbon and hydrogen, respectively.

1. **The repeat adsorption tests.**

The repeated tests of GN and B1 were performed for the same measurements, as shown in Fig. S2. And the data with the standard deviations have been added to the data. The data is consistent with that in main text which means the repeatability of the experiment is good.


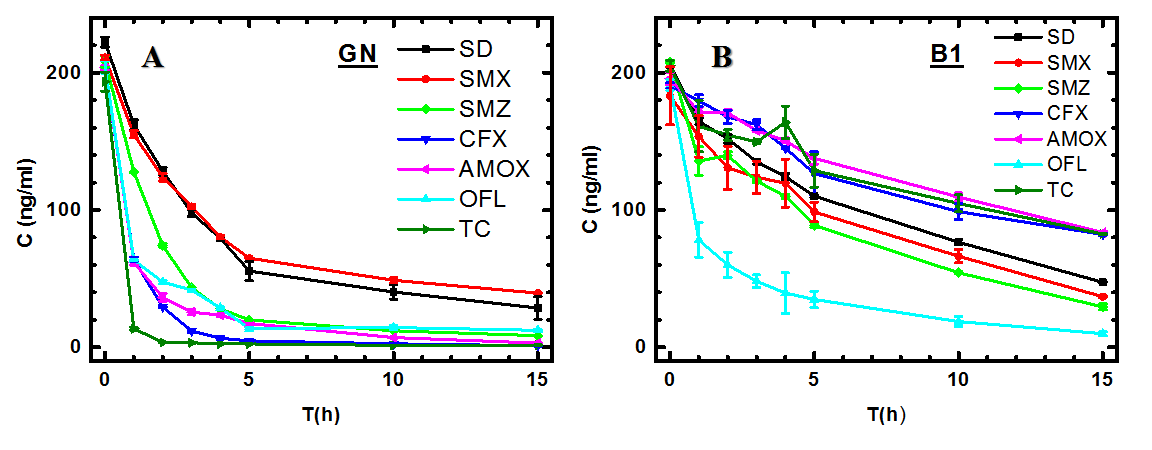


**Figure S2.** The change of the concentration of antibiotics in solution with time for graphene(GN) and coconut shell biochar(B1).

**REFERENCES**

1. Novak, J. M.; Busscher, W. J.; Laird, D. L.; Ahmedna, M.; Watts, D. W.; Niandou, M. A. S., Impact of Biochar Amendment on Fertility of a Southeastern Coastal Plain Soil. Soil Sci. 2009, 174, (2), 105-112.

2. Ditchfield, R.; Spencer, T. A., Carbocation-pi interaction: the 1,1-dimethylallyl cation and benzene. Tetrahedron Letters 2011, 52, (28), 3674-3677.

3. Peng, C. Y.; Ayala, P. Y.; Schlegel, H. B.; Frisch, M. J., Using redundant internal coordinates to optimize equilibrium geometries and transition states. Journal Of Computational Chemistry 1996, 17, (1), 49-56.

4. Frisch, M.; Trucks, G.; Schlegel, H.; Scuseria, G.; Robb, M.; Cheeseman, J.; Scalmani, G.; Barone, V.; Mennucci, B.; Petersson, G., Gaussian 09, revision A. 1. Gaussian Inc., Wallingford, CT 2009.
